# GNA14 may be a potential prognostic biomarker in nasopharyngeal carcinoma

**DOI:** 10.3389/fonc.2024.1482038

**Published:** 2024-11-26

**Authors:** Jinrong Hao, Xia Yu, Lei Xiong, Li Deng, Shifeng Lian, Shijun Sun, Xiaoling Li, Yun Du, Mingfang Ji

**Affiliations:** ^1^ Cancer Research Institute of Zhongshan City, Zhongshan City People’s Hospital, Zhongshan, China; ^2^ Department of Pathology, Zhongshan City People’s Hospital, Zhongshan, China; ^3^ Department of Oncology, Southwest Hospital, Army Medical University, Chongqing, China

**Keywords:** nasopharyngeal carcinoma, biomarker, GNA14, ebv, prognosis, immunohistochemistry, bioinformatics analysis

## Abstract

**Background:**

Nasopharyngeal carcinoma (NPC) is a highly invasive malignant tumor. Recurrence and distant metastasis represent the primary causes of treatment failure. This study aimed to identify biomarkers highly associated with NPC and investigate its roles in tumor progression.

**Methods:**

Transcriptome sequencing (RNA-seq) data of NPC and normal tissues were downloaded from the Gene Expression Omnibus (GEO) database. By analyzing the RNA-seq data, we found that G Protein Subunit Alpha 14 (GNA14) is closely associated with the diagnosis and prognosis of NPC. Immunohistochemistry (IHC) was used to detect the expression of GNA14 in tumor tissues of 165 NPC patients, and we analyzed the relationship between GNA14 expression and patient prognosis. The potential mechanisms by which GNA14 affects tumor prognosis were preliminarily analyzed using bioinformatics analysis.

**Results:**

Analysis of RNA-seq data and IHC showed that GNA14 expression was downregulated in NPC (*p* < 0.001, *p* < 0.01, respectively), and low expression of GNA14 was closely associated with poor prognosis. IHC analysis showed that patients with low GNA14 expression had significantly shorter progression-free survival (PFS) and distant metastasis-free survival (DMFS) than those with high GNA14 expression (*p* = 0.023, *p* = 0.008, respectively). Multivariate analysis indicated that the GNA14 expression was an independent risk factor for DMFS (*p* = 0.030). The DMFS nomogram included GNA14 expression, EBV DNA, and N stage as prognostic factors and the concordance index (C-index) of the nomogram was 0.73. Bioinformatics analysis indicated that NPC patients with low GNA14 expression might represent lower levels of immune cell infiltration and poorer drug sensitivity.

**Conclusion:**

Low GNA14 expression may be a risk factor for poor prognosis in NPC.

## Introduction

Nasopharyngeal carcinoma (NPC), a type of head and neck squamous cell carcinoma (HNSCC), originates from nasopharyngeal epithelial tissues (1). NPC is characterized by an uneven geographical distribution, particularly prevalent in East and Southeast Asia (2) The etiology of the disease is related to racial susceptibility, genetic factors, environmental factors, and Epstein-Barr virus (EBV) infection (1). In terms of treatment, NPC is treated by a combined regimen of radiotherapy and chemotherapy. This regimen has achieved significant success, with a marked increase in the survival rate for patients (3). However, recurrence and distant metastases remain the leading causes of death for NPC patients (2). Therefore, identifying patients at high risk of recurrence and metastasis before treatment can help oncologists develop individualized treatment plans, which are essential for improving outcomes and prolonging the survival of NPC patients. A recent study proposes that NPC should be viewed as a multidimensional spatiotemporal “unity of ecology and evolution” pathological ecosystem (4). The ecological theory of NPC suggests that tumor cells behave like invasive species in a dynamic ecosystem, interacting with the tumor microenvironment (TME), the immune system, and various factors. It is emphasized that certain genes are not only markers of disease but also key players in ecological interactions in the tumor microenvironment. For example, genes associated with immune evasion, cell proliferation, and metastasis contribute to the enhanced invasiveness of NPC. Therefore, identifying such key biomarkers is crucial for elucidating the molecular mechanisms of recurrence and distant metastasis in NPC.

Identifying biomarkers closely associated with NPC and exploring their value in the diagnosis and treatment of the tumor is currently a major research focus (5, 6). RNA-seq technology has been extensively applied in NPC research to elucidate the association between biomarkers and disease mechanisms, tumor biology, and prognosis (7). Against this backdrop, machine learning algorithms and bioinformatics technologies have played a pivotal role. Advances in bioinformatics enable comprehensive analysis of RNA-seq data (8). The combination of bioinformatics analysis and machine learning algorithms has facilitated the identification of biomarkers highly associated with tumors (9). Identifying key biomarkers in tumors is crucial for developing personalized treatment strategies and understanding the molecular mechanisms of NPC. For instance, in the treatment of NPC, outcomes are often influenced by the unique immune evasion mechanisms of the tumor. Recent studies have identified that certain key biomarkers influence the number and proportion of immune cells in the TME, thereby affecting the therapeutic outcomes for patients (10, 11). By analyzing the characteristics of immune infiltration associated with these biomarkers, new avenues for targeted therapies can be identified (12).

The prognosis of NPC primarily depends on the extent of tumor infiltration. However, in addition to the American Joint Committee on Cancer (AJCC) tumor-node-metastasis (TNM) staging, various other prognostic factors, including age, gender, smoking history, EBV DNA load, and gene expression levels (13–15), have been observed. Gene Expression Omnibus (GEO) and The Cancer Genome Atlas (TCGA) provide a wealth of RNA-seq data and corresponding clinical information (16, 17), which play a crucial role in the discovery of new functional genes and understanding of the pathogenesis of tumors. In this study, NPC-related RNA-seq data were analyzed using bioinformatics methods, identifying that GNA14 was strongly associated with the prognosis of NPC. Immunohistochemistry was used to verify the prognostic value of GNA14 in clinical samples. The intrinsic association between GNA14 and NPC was investigated by functional enrichment analysis, immune infiltration analysis, drug sensitivity analysis, and other methods.

## Materials and methods

### Raw data acquisition and preprocessing

RNA-seq datasets related to NPC (GSE12452, GSE53819, GSE64634, GSE61218, GSE102349) were downloaded from the GEO database. Raw data were log-transformed and normalized using the “limma” software package. Expression data of the same genes in the GSE12452 and GSE53819 were merged to form a merged expression matrix, which was subjected to batch effect removal using the “sva” package to generate a training set for the screening of key biomarkers. Using the same method, we merged GSE64634 and GSE61218 as a validation set. The GSE102349 dataset contains clinical and survival information from 113 NPC patients, which was used for survival analysis and subsequent bioinformatics analysis. RNA-seq data and clinical information from 33 solid tumors and corresponding normal tissues were downloaded from the TCGA database for the subsequent pan-cancer analysis. The characteristics of the GEO datasets and the tumor types in the TCGA database are summarized in **Supplementary Table 1**.

### Differential expression analysis and machine learning algorithms

Differential expression analysis was performed on the training set using the “limma” package, with |log2FC| > 1 and false discovery rate (FDR) < 0.05 as screening criteria for identifying differentially expressed genes (DEGs) (18). The DEGs were further screened utilizing Random Forest (RF), Least Absolute Shrinkage and Selection Operator (LASSO) logistic regression, and Support Vector Machine-Recursive Feature Elimination (SVM-RFE) algorithms. RF was performed using the “randomForest” package, genes with a score > 1 were considered as key genes based on the “importance” function. The LASSO regression model was constructed utilizing the “glmnet” package (19) for variable selection. The cross-validation method was used to determine the optimal lambda value corresponding to the number of key genes. SVM-RFE was performed using the “e1071” package to conduct 10-fold cross-validation and feature elimination, identifying a set of key genes based on the principle of error minimization.

### Selection of the key biomarker

The overlapping genes identified by the three algorithms were considered highly related to NPC. The diagnostic value of these genes was assessed by receiver operating characteristic curve (ROC) analysis and validated through the validation set. 88 patients from GSE102349 with complete progression-free survival (PFS) information were used for survival analysis. The RNA-seq data and PFS information from the TCGA were used to conduct a pan-cancer survival analysis. Grouping for survival analysis was based on median expression levels of the overlapping genes (GNA14 and LRRC34). Through the above process, we successfully identified a key biomarker, GNA14, highly related to the diagnosis and prognosis of NPC.

### Study population in hospital and follow-up

Tissue specimens for immunohistochemical (IHC) examination of GNA14 were obtained from 165 diagnosed NPC patients treated at Zhongshan City People’s Hospital (Guangdong, China) from January 2015 to December 2017, along with 30 patients diagnosed with chronic rhinosinusitis during the same period. This study was conducted in compliance with the Declaration of Helsinki. The study received approval from the Clinical Research Ethics Committee of the Zhongshan City People’s Hospital. All NPC tissues were collected before anti-cancer treatment. All patients following these criteria were retrospectively enrolled: (a) histopathologically confirmed NPC; (b) clinical stages I-IVa according to the 8th edition AJCC/UICC staging system; (c) received either solely intensity-modulated radiation therapy, concurrent chemoradiotherapy, with or without induction chemotherapy or adjuvant chemotherapy; (d) had complete baseline data; (e) had no severe heart, lung, liver, kidney diseases, or other cancers at NPC diagnosis. NPC patients who completed treatment were followed monthly for the first 3 months, every 3 months for the next 3 years, every 6 months for the next 2 years, and annually thereafter. Follow-up ended in December 2023.

### IHC and scoring strategies

All Tissues were fixed in 4% formaldehyde and embedded in paraffin. To assess the expression level of GNA14, IHC examinations were conducted using the GNA14 antibody (Polyclonal, rabbit, 13350-1-AP-50UL, 1:200 dilution, Wuhan Sanying). Tissues were cut into 4 μm sections, deparaffinized with xylene, and subsequently rehydrated with graded ethanol. The slides were then incubated with a 3% H2O2 solution for 10 minutes to quench endogenous peroxidase activity, and 0.01 mmol/L citrate buffer (pH 6.0) was used for antigen retrieval in a high-pressure cooker. The sections were incubated with the primary antibody for 3 hours at room temperature, followed by rinsing with TBS. Following incubation with the secondary antibody (Anti-Mouse/Rabbit Universal Immunohistochemical Test Kit, PK10006, Wuhan Sanying), the sections were stained with DAB. All slides were then re-stained with hematoxylin, examined under the microscope, and photographed. Positive control sections were provided by the antibody manufacturer. Cell staining intensity was scored based on a previous study (20, 21). The IHC results were assessed by calculating the total score (0-12) by multiplying the intensity of positive staining (negative, 0; weak, 1; moderate, 2; or strong, 3) by the proportion of target immunopositive cells (<25%, 1; 25-49%, 2; 50-75%, 3; or >75%, 4). IHC results were evaluated independently by two pathologists, and any discrepancies were resolved through consensus. Based on the median IHC score, the high GNA14 expression group was defined as samples with an IHC score > 4, and the low GNA14 expression group was defined as samples with an IHC score ≤ 4.

### Expression profile of GNA14

RNA-seq data were extracted from the training and validation sets to compare the expression levels of GNA14 in NPC and normal tissue. IHC staining was employed to detect and compare the expression of GNA14 in nasopharyngeal tissues from patients with chronic sinusitis and NPC. Furthermore, we assessed the differential expression of GNA14 in multiple solid tumors by analyzing RNA-Seq data from the TCGA database, covering 33 different types of tumors and their corresponding normal tissues.

### Functional enrichment analysis

Based on the median GNA14 expression level, NPC samples in GSE102349 were categorized into high and low GNA14 expression groups. Identification of DEGs between two groups using the “limma” package (|log2FC| >1, FDR < 0.05). Gene Ontology (GO) and Kyoto Encyclopedia of Genes and Genomes (KEGG) pathways of the DEGs were performed using the “clusterProfiler” package. We selected “C5.go.Hs.symbols.gmt” from the Molecular Signatures Database (MSigDB) as the reference gene set, and gene set enrichment analysis (GSEA) was performed for genes in the GNA14 high and low expression groups.

### Immune infiltration and drug sensitivity analysis

The “estimate” package in R was employed to predict the ImmuneScore, StromalScore, and their combined scores (ESTIMATE score) in tumor samples. Expression levels of GNA14 and 79 immune checkpoint genes were extracted, followed by Spearman correlation analysis to evaluate the association between GNA14 and each immune checkpoint gene. Genes with a p-value less than 0.001 were identified as immune checkpoint genes highly associated with GNA14. The list of immune checkpoint genes was derived from previously published literature (22). The single-sample gene set enrichment analysis (ssGSEA) algorithm was utilized to assess the relationship between the proportions of various immune cell types in NPC and GAN14 expression. The gene annotation file contains 28 tumor-infiltrating immune cells from TISIDB. Furthermore, the “oncoPredict” package was used to estimate the chemotherapeutic response of patients from high and low GNA14 groups. The chemotherapeutic response was determined by the half maximal inhibitory concentration (IC50) of each NPC patient and the IC50 data was sourced from the GDSC website (https://www.cancerrxgene.org/).

### Statistical analysis

Descriptive statistical analysis was performed on the collected data, expressed as mean ± standard deviation (SD) or percentage (%). Continuous variables were compared using the independent samples t-test or Mann-Whitney U test. Correlations between variables were assessed using Pearson or Spearman correlation analysis. The division of high and low GNA14 expression groups was based on the median GNA14 expression level. Survival analysis was performed using the Kaplan-Meier method, and log-rank tests were used to compare differences. Correlations between GNA14 expression and clinicopathological features were analyzed using the chi-square test or Fisher exact test. The cutoff value for the high and low EBV DNA groups was chosen as 4000 copies per milliliter based on a previous study of the prognostic value of EBV DNA (23). Univariate and multivariate Cox regression models were used to identify the risk factors associated with the prognosis of NPC. A nomogram was constructed to predict the 3-year and 5-year DMFS of NPC patients based on the results of multivariate analysis, and the predictive ability of the models was assessed by calculating the consistency index (C-index). All tests were two-tailed, with significance levels set at *p* < 0.05. All statistical analyses were performed using R software (version 4.0.0) or SPSS software (version 20.0, IBM, New York, USA).

## Results

### Differential expression analysis and machine learning algorithms based on GEO datasets

**Figure 1:**
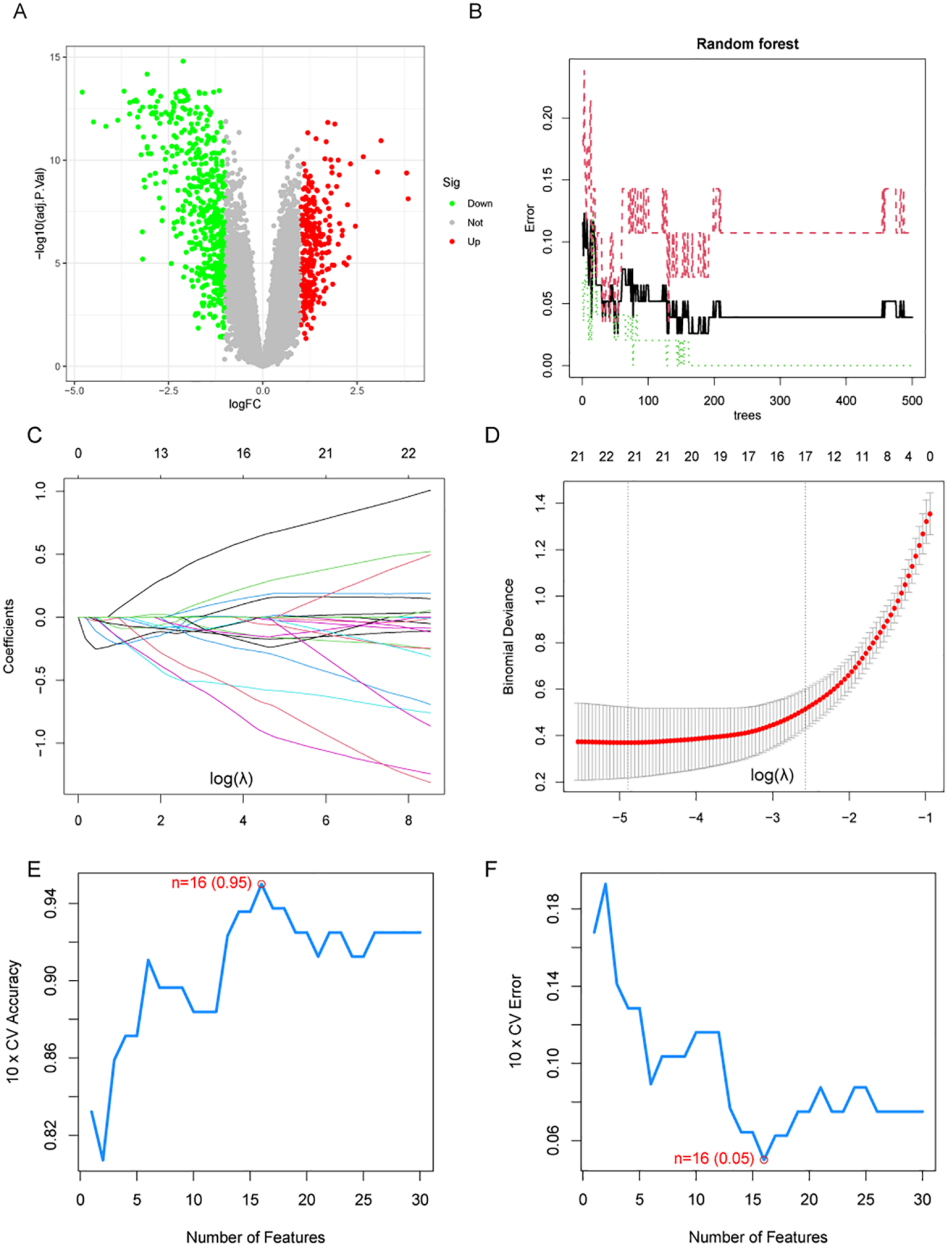
Differential expression analysis and machine learning algorithms. **(A)** Volcano plot shows the differentially expressed genes (DEGs) in NPC and normal tissues. **(B)** The relationship between the number of decision trees and the error rate in the random forest algorithm. **(C, D)** LASSO regression analysis. **(E, F)** Accuracy and error curves for feature variables in the SVM-RFE.

A total of 795 DEGs were identified between NPC and normal tissues based on GEO datasets (**Supplementary Table 2**). Among these genes, 515 were up-regulated and 280 were down-regulated in NPC samples (**Figure 1A**). Using the RF algorithm, LASSO regression algorithm, and SVM-RFE algorithm, 4, 21, and 16 genes were identified as highly associated with NPC, respectively (**Figures 1B–F**, **Supplementary Table 3**).

Differential expression analysis and machine learning algorithms based on GEO datasets.

### GNA14 may be a key biomarker in NPC

**Figure 2:**
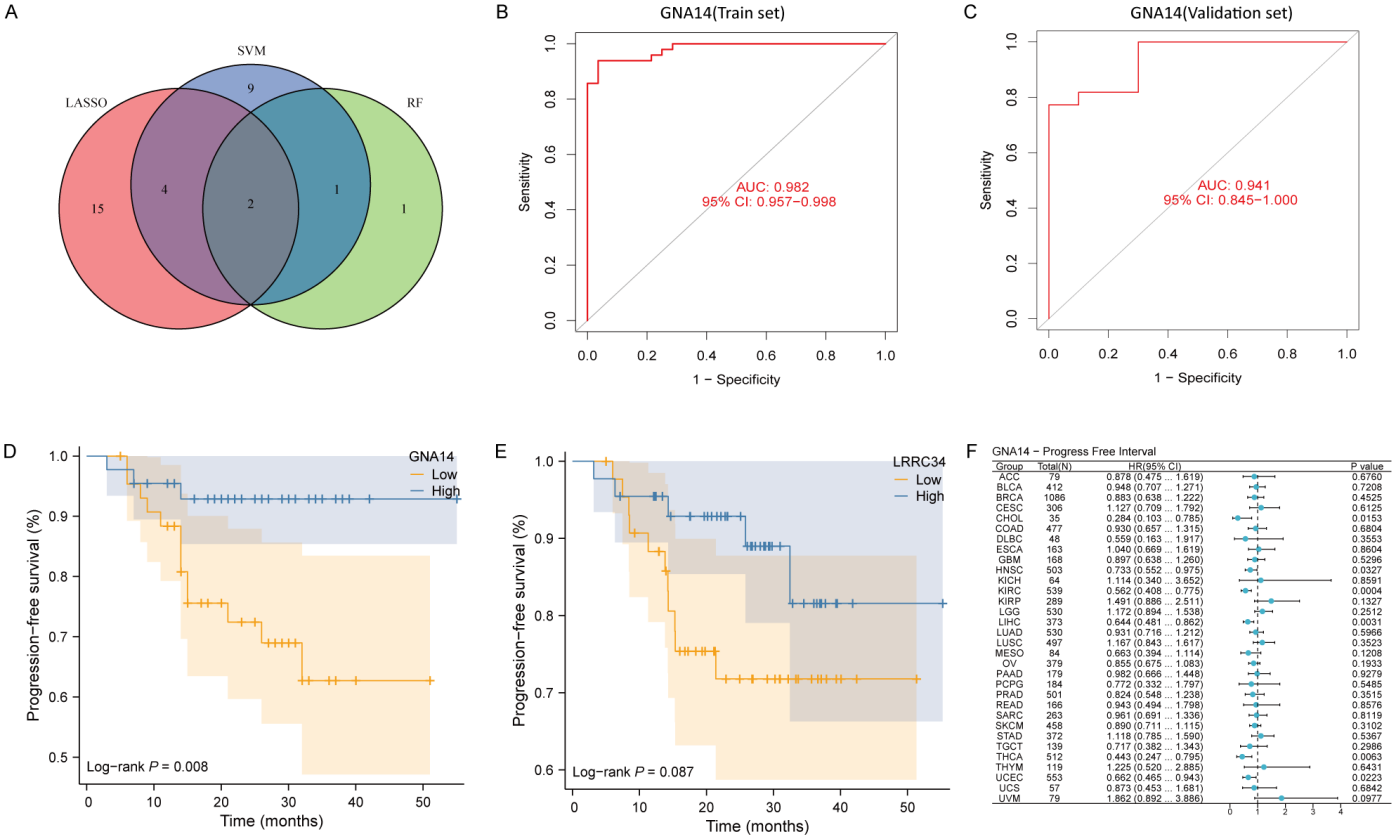
GNA14 may be a key biomarker in NPC. **(A)** Venn diagram showing the overlapped genes from machine learning algorithms. **(B, C)** ROC curve of GNA14 in the training set and validation set, with the horizontal axis representing the false positive rate (FPR) and the vertical axis indicating sensitivity. **(D)** Kaplan-Meier curves of PFS for 88 patients between high GNA14 expression group and low GNA14 expression group. **(E)** Kaplan-Meier curves of PFS for patients between high LRRC34 expression group and low LRRC34 expression group. **(F)** Pan-cancer survival analysis between high and low GNA14 expression groups.

The Venn diagram showed two overlapping genes (GNA14, LRRC34) identified by three machine learning algorithms (**Figure 2A**, **Supplementary Table 2**). ROC curve analysis demonstrated that GNA14 had an AUC of 0.982 (95% CI: 0.96-0.99) in the training set (**Figure 2B**), and achieved an AUC of 0.941 (95% CI: 0.85-1.00) in the validation set (**Figure 2C**). The results of the survival analysis indicated that the group with low GNA14 expression exhibited significantly shorter PFS compared to the high-expression group (**Figure 2D**). Conversely, there were no significant differences in PFS between the high-expression and low-expression groups of LRRC34 (**Figure 2E**). In addition, we found that in head and neck squamous cell carcinoma (HNSCC), thyroid carcinoma (THCA), cholangiocarcinoma (CHOL), kidney renal clear cell carcinoma (KIRC), liver hepatocellular carcinoma (LIHC), and uterine corpus endometrial carcinoma (UCEC), the risk of tumor progression was higher in the GNA14 low-expression group than in the GNA14 high-expression group (**Figure 2F**). Therefore, GNA14 was selected as a key biomarker for further study.

### Expression profile of GNA14

**Figure 3:**
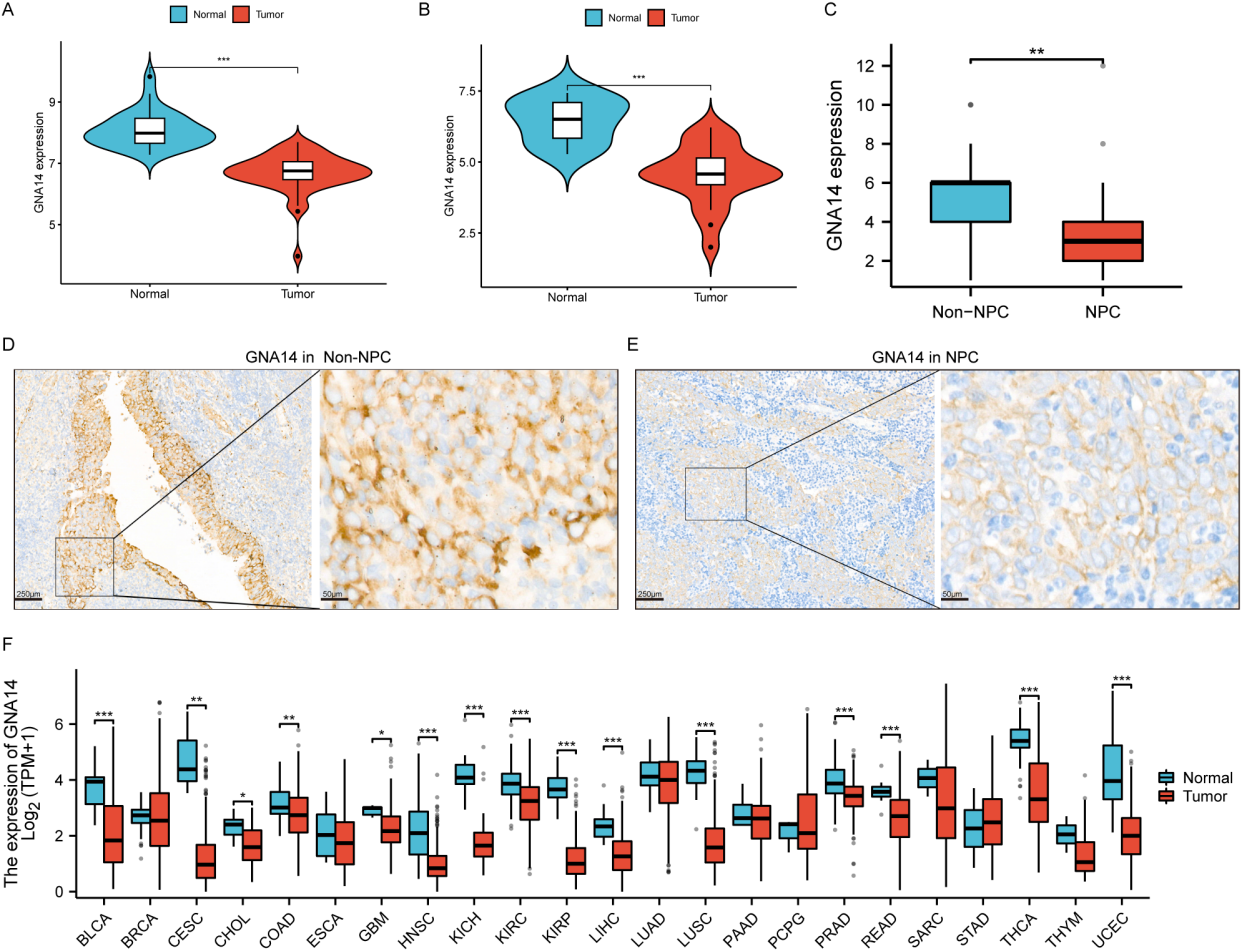
GNA14 expression profile. **(A, B)** Box plot showing differential expression of GNA14 in normal nasopharyngeal mucosa and NPC tissues, with the left graph representing the training set and the right graph representing the validation set. **(C)** Box plot showing differential expression of GNA14 in chronic rhinosinusitis tissues (Non-NPC) and NPC tissues (Based on IHC results). **(D, E)** IHC examination of GNA14 expression in NPC tissues and in non-NPC tissues [magnification 40x (left) and 200x (right)]. **(F)** Differential expression analysis was conducted on GNA14 in various tumor samples and normal controls (*** *p* < 0.001, ** *p* < 0.01, * *p* < 0.05).

Analysis of the RNA-seq data demonstrated a significant reduction in GNA14 expression in NPC compared to normal nasopharyngeal tissues in training set (*p* < 0.001) (**Figure 3A**), a result corroborated by the validation set (**Figure 3B**). Through IHC examination, we observed that the expression level of GNA14 in NPC samples was significantly lower than in non-cancerous nasopharyngeal tissues (*p* < 0.01), and GNA14 was primarily localized in the cell membrane (**Figures 3C–E**). Furthermore, it was discovered that in various tumor samples, such as head and neck squamous cell carcinoma (HNSCC), bladder urothelial carcinoma (BLCA), liver hepatocellular carcinoma (LHIC), lung squamous cell carcinoma (LUSC), and thyroid carcinoma (THCA), the expression level of GNA14 was significantly lower than that of the corresponding normal samples (*p* < 0.001) (**Figure 3F**).

### Patient characteristics and clinical sample analysis

**Figure 4:**
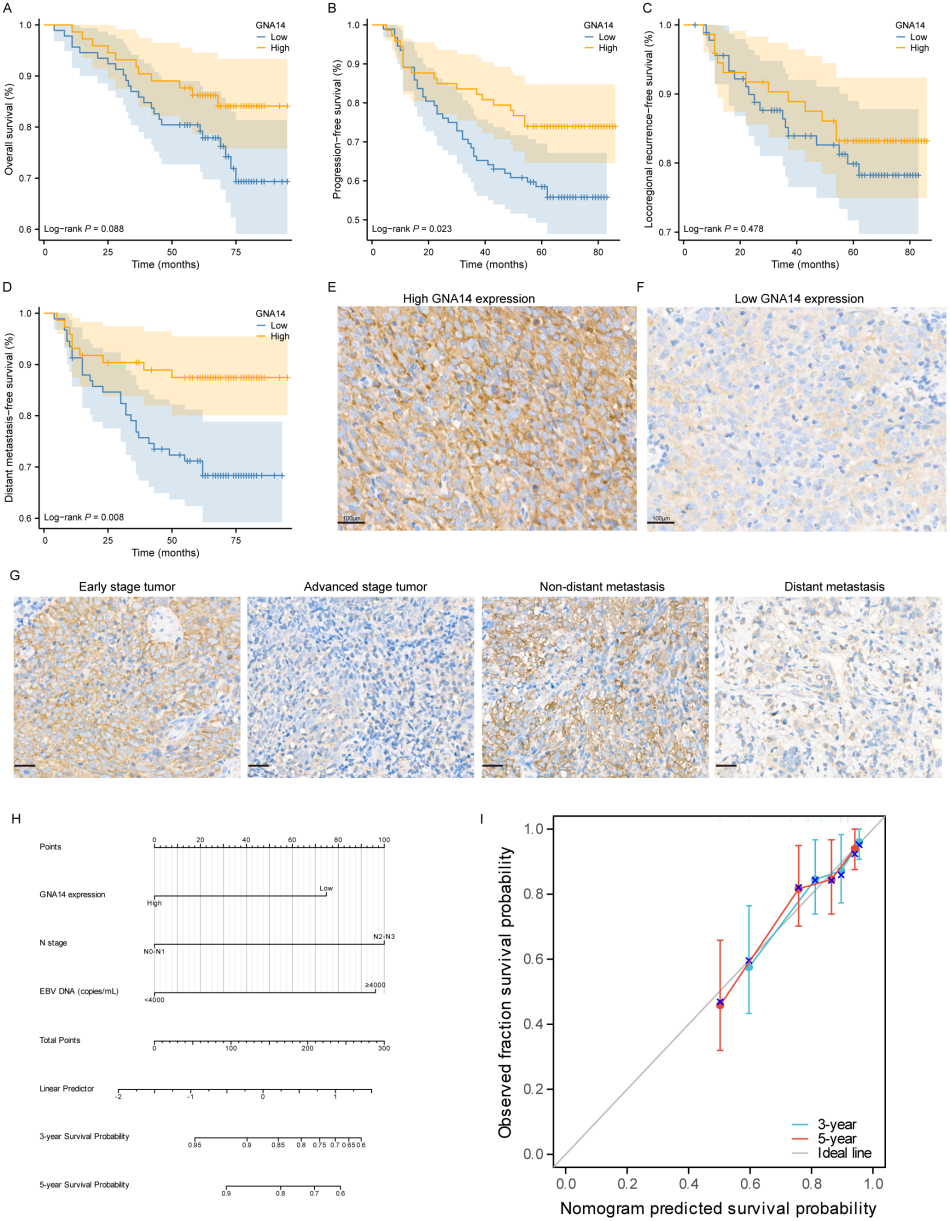
Low expression of GNA14 predicts poor prognosis in NPC patients. Kaplan–Meier curves of OS **(A)**, PFS **(B)**, LFRS **(C)**, and DMFS **(D)** for high and low GNA14 expression in NPC patients. **(E, F)** Representative images of high and low expression groups of GNA14 immunohistochemical staining in NPC tissue (100×). **(G)** Representative images of GNA14 expression in the clinical staging group and the distant metastasis group. **(H)** The nomogram was used to predict the 3-year and 5-year DMFS rates for patients with NPC. **(I)** The calibration curve for the nomogram.

Of the 165 patients enrolled, 110 (66.7%) were male and 55 (33.3%) were female, with a male-to-female ratio of 2:1, and a median age of 49 years (range: 22-76 years). 39 (23.6%) patients were diagnosed as stage I-II and 126 (76.4%) as stage III-IV. The majority of patients had a pathological diagnosis of WHO type III (97.5%). The median duration of follow-up was 69 months (range: 4-95). There were 92 cases in the GNA14 low expression group and 73 cases in the GNA14 high expression group (**Figures 4E, F**). We found that the low GNA14 expression was significantly associated with advanced clinical stage (*p* = 0.013) and increased risk of distant metastasis (*p* = 0.008) (**Table 1**, **Figure 4G**). The PFS (56.5% vs. 74.0%; *p* = 0.023) and DMFS (70.0% vs. 87.7%; *p* = 0.008) of patients in the GNA14-low expression group were significantly shorter than those in the GNA14-high expression group (**Figures 4A, B**), while there was no significant difference in overall survival (OS) and locoregional recurrence-free survival (LRFS) between the two groups (*p* = 0.088, *p* = 0.478, respectively) (**Figures 4C, D**). The results of the multivariate Cox regression analysis indicated that T stage and EBV DNA were significant factors for OS (*p* = 0.024 and *p* = 0.029, respectively). EBV DNA was identified as a significant factor for PFS (*p* = 0.012), while T stage was a significant factor for LRFS (*p* = 0.031). GNA14 expression was an independent risk factor affecting DMFS in NPC (*p* = 0.030) (**Table 2**). We constructed a nomogram that incorporates the GNA14 expression (**Figures 4H, I**), EBV DNA, and N stage to predict the 3-year and 5-year DMFS in NPC patients (**Figure 4G**). The C-index of the nomogram was 0.73 (**Figure 4H**).

**Table 1:** Baseline information of enrolled patients.

Characteristics	GNA14 expression		P value	overall
	Low	High		
n	92	73		165
Sex, n (%)			0.912	
female	31 (33.7%)	24 (32.9%)		55 (33.3%)
male	61 (66.3%)	49 (67.1%)		110 (66.7%)
Smoking, n (%)			0.059	
No	75 (81.5%)	67 (91.8%)		142 (86.1%)
Yes	17 (18.5%)	6 (8.2%)		23 (13.9%)
Age, n (%)			0.356	
<60	73 (79.3%)	62 (84.9%)		135 (81.8%)
≥60	19 (20.7%)	11 (15.1%)		30 (18.2%)
T.Stage, n (%)			0.304	
T1-T2	43 (46.7%)	40 (54.8%)		83 (50.3%)
T3-T4	49 (53.3%)	33 (45.2%)		82 (50.7%)
N.Stage, n (%)			0.067	
N0-N1	36 (39.1%)	39 (53.4%)		75 (45.5%)
N2-N3	56 (60.9%)	34 (46.6%)		90 (54.5%)
TNM.Stage, n (%)			**0.013**	
I-II	15 (16.3%)	24 (32.9%)		39 (23.6%)
III-IV	77 (83.7%)	49 (67.1%)		126 (76.4%)
EBV DNA (copies/mL), n (%)			0.201	
≥4000	47 (51.1%)	30 (41.1%)		77 (46.7%)
<4000	45 (48.9%)	43 (58.9%)		88 (53.3%)
Death, n (%)			0.086	
No	68 (73.9%)	62 (84.9%)		130 (78.8%)
Yes	24 (26.1%)	11 (15.1%)		35 (21.2%)
Recurrence, n (%)			0.605	
No	74 (80.4%)	61 (83.6%)		135 (81.8%)
Yes	18 (19.6%)	12 (16.4%)		30 (18.2%)
Distant metastasis, n (%)			**0.006**	
No	64 (69.6%)	64 (87.7%)		128 (77.6%)
Yes	28 (30.4%)	9 (12.3%)		37 (22.4%)
WHO type, n (%)			0.457	
III	91 (98.9%)	70 (95.9%)		161 (97.6%)
I	1 (1.1%)	3 (4.1%)		4 (2.4%)

Bold values indicate statistically significant results (p < 0.05).

**Table 2:** Univariate and multivariate analysis of prognostic risk factors in NPC patients.

Characteristics	Hazard ratio (95% CI)	P value	Hazard ratio (95% CI)	P value
OS	Univariate analysis		Multivariate analysis	
GNA14 expression (high vs. low)	0.542 (0.266 - 1.107)	0.093	0.619 (0.299 - 1.279)	0.195
Sex (Female vs. male)	1.222 (0.586 - 2.544)	0.593		
Age (<60 vs. ≥60)	2.207 (1.081 - 4.509)	**0.030**	1.856 (0.899 - 3.832)	0.095
Smoking (No vs. Yes)	1.357 (0.562 - 3.275)	0.497		
T.Stage (T1-T2 vs. T3-T4)	3.324 (1.557 - 7.096)	**0.002**	3.045 (1.160 - 7.996)	**0.024**
N.Stage (N0-N1vs. N2-N3)	1.746 (0.868 - 3.511)	0.118		
TNM.Stage (I-II vs. III-IV)	2.669 (0.942 - 7.562)	0.065	0.794 (0.202 - 3.116)	0.741
EBV DNA (<4000 vs.≥4000) (copies/mL)	2.729 (1.336 - 5.573)	**0.006**	2.283 (1.086 - 4.799)	**0.029**
PFS	Univariate analysis		Multivariate analysis	
GNA14 expression (high vs. low)	1.866 (1.080 - 3.224)	**0.025**	1.705 (0.973 - 2.987)	0.062
Sex (Female vs. male)	2.207 (1.170 - 4.162)	**0.014**	1.850 (0.957 - 3.577)	0.067
Age (<60 vs. ≥60)	1.554 (0.864 - 2.793)	0.141		
Smoking (No vs. Yes)	0.976 (0.463 - 2.058)	0.950		
T.Stage (T1-T2 vs. T3-T4)	1.479 (0.884 - 2.474)	0.136		
N.Stage (N0-N1vs. N2-N3)	1.957 (1.133 - 3.381)	**0.016**	1.226 (0.621 - 2.421)	0.557
TNM.Stage (I-II vs. III-IV)	2.330 (1.105 - 4.912)	**0.026**	1.202 (0.474 - 3.050)	0.698
EBV DNA (<4000 vs. ≥4000) (copies/mL)	2.530 (1.483 - 4.314)	**< 0.001**	2.041 (1.173 - 3.551)	**0.012**
DMFS	Univariate analysis		Multivariate analysis	
GNA14 expression (high vs. low)	2.677 (1.263 - 5.675)	**0.010**	2.330 (1.084 - 5.010)	**0.030**
Sex (Female vs. male)	2.275 (0.999 - 5.179)	0.050	1.597 (0.684 - 3.728)	0.279
Age (<60 vs. ≥60)	1.475 (0.696 - 3.126)	0.311		
Smoking (No vs. Yes)	0.523 (0.161 - 1.703)	0.282		
T.Stage (T1-T2 vs. T3-T4)	0.918 (0.481 - 1.752)	0.795		
N.Stage(N0-N1vs. N2-N3)	4.033 (1.770 - 9.188)	**< 0.001**	3.590 (1.084 - 11.894)	**0.037**
TNM.Stage (I-II vs. III-IV)	2.880 (1.020 - 8.132)	**0.046**	0.513 (0.112 - 2.343)	0.389
EBV DNA (<4000 vs. ≥4000) (copies/mL)	3.645 (1.763 - 7.537)	**< 0.001**	2.772 (1.301 - 5.905)	**0.008**
LRFS	Univariate analysis		Multivariate analysis	
GNA14 expression (high vs. low)	1.300 (0.626 - 2.701)	0.481		
Sex (Female vs. male)	2.704 (1.035 - 7.067)	**0.042**	2.561 (0.978 - 6.702)	0.055
Age (<60 vs. ≥60)	1.734 (0.771 - 3.898)	0.183		
Smoking (No vs. Yes)	2.042 (0.875 - 4.763)	0.099		
T.Stage (T1-T2 vs. T3-T4)	2.420 (1.132 - 5.174)	**0.023**	2.309 (1.079 - 4.943)	**0.031**
N.Stage (N0-N1vs. N2-N3)	0.996 (0.486 - 2.041)	0.990		
TNM.Stage (I-II vs. III-IV)	1.771 (0.678 - 4.627)	0.244		
EBV DNA (<4000 vs. ≥4000) (copies/mL)	1.733 (0.841 - 3.570)	0.136		

Bold values indicate statistically significant results (p < 0.05).

### Functional enrichment analysis

**Figure 5:**
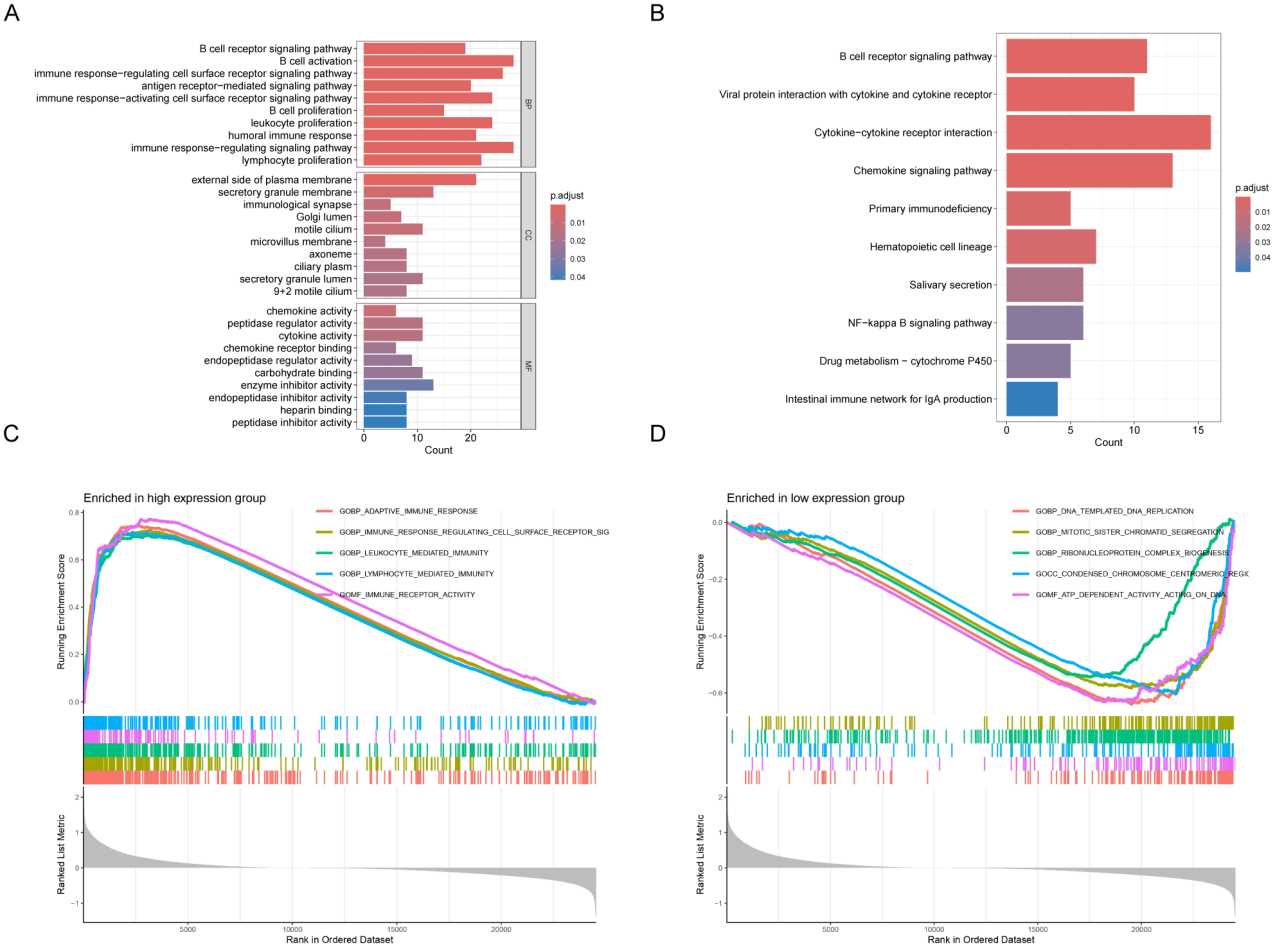
Analysis of functional enrichment in GNA14 high and low expression groups in 113 NPC patients from GSE102349. Gene Ontology **(A)** and KEGG pathways **(B)**. Results of differentially expressed genes (DEGs) in patients with high and low GNA14 expression. GSEA analysis revealed gene pathways that were significantly enriched in the high **(C)** and low **(D)** GNA14 expression groups.

We identified 280 DEGs between high and low GNA14 expression groups in GSE102349, with 266 genes exhibiting upregulation and 14 showing downregulation (**Supplementary Table 4**). GO analysis indicated that these DEGs were predominantly enriched in pathways related to immune response and cell migration, including B-cell activation, proliferation of various immune cells, and ciliary movement (**Figure 5A**). KEGG enrichment analysis revealed that the DEGs were significantly concentrated in pathways such as chemokine signaling, NF-kB signaling, and cytochrome P450-mediated drug metabolism (**Figure 5B**). According to Gene Set Enrichment Analysis (GSEA), pathways related to cell division, DNA, and chromosome replication were up-regulated in the low GNA14 expression group, and pathways related to immune cell activity and adaptive immune response were down-regulated compared to patients in the high GNA14 expression group (**Figures 5C, D**).

### Immune cell infiltration and drug sensitivity analysis

**Figure 6:**
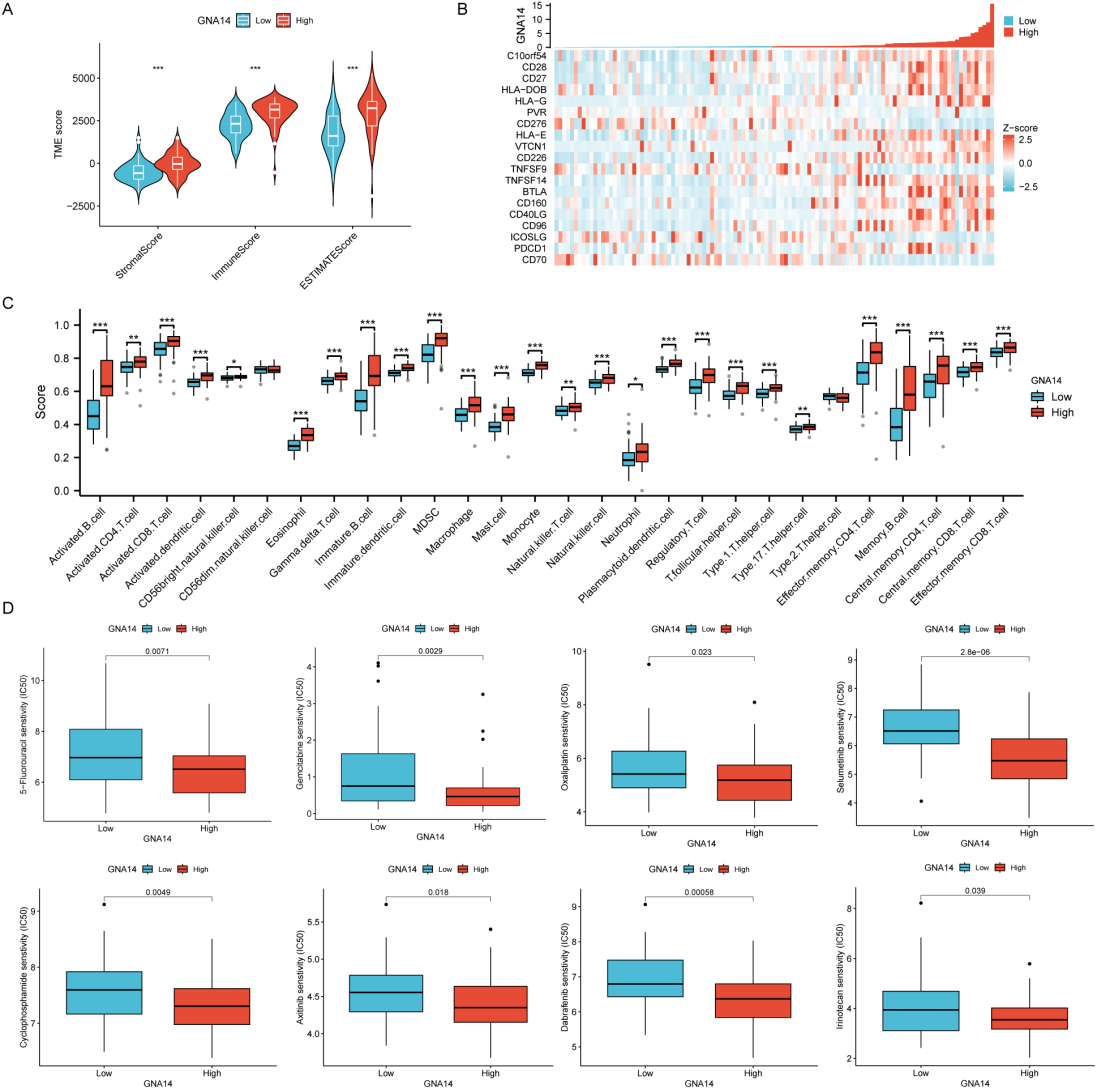
Immune cell infiltration and drug sensitivity analysis in 113 NPC patients from GSE102349. **(A)** Box plots were used to compare the differences between GNA14 high and low expression groups in terms of immunity score, stroma score, and estimate score. **(B)** Correlation between GNA14 expression levels and immune checkpoint genes. **(C)** The levels of multiple immune cell counts were compared between high and low GNA14 expression groups (*** *p* < 0.001, ** *p* < 0.01, * *p* < 0.05). **(D)** Box plots were used to compare drug sensitivity between GNA14 high and low expression groups, the horizontal axis indicates the drug name and the vertical axis indicates drug sensitivity (IC50).

It was found tumor tissues with low GNA14 expression represented lower immune and stromal scores (**Figure 6A**). Furthermore, in NPC patients with low GNA14 expression, the majority of immune checkpoint genes exhibit lower expression levels (*p* < 0.001) (**Figure 6B**, **Supplementary Table 5**). Results from the immune infiltration analysis showed that a decrease in GNA14 expression level was correlated with a reduced proportion of most immune cells such as B cells, CD8 T cells, and NK cells (**Figure 6C**). Through drug sensitivity analysis, we observed that most chemotherapy drugs such as 5-fluorouracil, Gemcitabine, and Oxaliplatin exhibited higher IC50 values in patients with GNA14-low expression (**Figure 6D**).

## Discussion

Exploring the key biomarkers that affect the prognosis of NPC is crucial for identifying new therapeutic targets and prolonging the survival of patients. In this study, through a comprehensive analysis of RNA-seq data, we ultimately identified GNA14 as a potential key biomarker for NPC. Subsequently, we performed IHC to examine the expression of GNA14 in NPC and to explore its correlation with the clinical characteristics of patients. Analysis of RNA-seq data and IHC analysis consistently showed that GNA14 expression was downregulated in NPC tissues.

GNA14 is a gene encoding the G protein subunit alpha 14. G proteins (guanine nucleotide-binding proteins) are widespread intracellular signal transduction molecules. Some studies have shown that GNA14 can act as an oncogene to promote cancer development. For example, a study by Wang et al. found that GNA14 is highly expressed in endometrial carcinoma tissue and that GNA14 promotes the malignant growth of endometrial carcinoma by stimulating KLF7 to upregulate HAS2 expression (24). Interestingly, more research indicates that GNA14 may function as a tumor suppressor gene For example, Song et al. found that GNA14 was downregulated in hepatocellular carcinoma (HCC), and negatively associated with hepatitis B virus (HBV) infection, vascular invasion, and HCC prognosis (25). Huang et al. reported lower GNA14 expression in oral squamous cell carcinoma (OSCC) cell lines and tissues, indicating a poorer prognosis for OSCC (26). In a study conducted by Pan et al., GNA14 exhibited low expression in papillary thyroid carcinoma and patients in the GNA14 low expression group demonstrated a diminished disease-free survival (DFS) rate (27). The function of GNA14 in tumor development may depend on the specific tumor type, with the protein exhibiting either tumor-promoting or tumor-suppressing properties. It is noteworthy that thyroid carcinoma, oral squamous cell carcinoma, and nasopharyngeal carcinoma are all clinically common head and neck tumors. Similarly, our study also found that GNA14 expression was down-regulated in NPC, and patients with low GNA14 expression had significantly lower PFS and DMFS than those with high GNA14 expression. Considering that the processing of the specimens might negatively affect the GNA14 measurements, we employed an antigen retrieval step in the experiment to ensure antibody binding to the target antigen. For instance, antigen retrieval was performed under high-pressure conditions using a 0.01 mmol/L citrate buffer (pH 6.0) in a pressure cooker. This approach has been demonstrated to restore the antigenicity of many proteins, ensuring reliable antibody binding (28, 29). Additionally, tissue fixation time or the extent of paraffin infiltration may compromise the accuracy of GNA14 quantification due to antigen masking. Therefore, we included positive control sections provided by the manufacturer, which helped to confirm the validity of the staining protocol.

In this study, we preliminarily investigated the possible reasons for the poor prognosis of NPC patients due to low GNA14 expression, as well as to explore the potential therapeutic approach of GNA14 as a target. It was observed that the expression level of GNA14 was lower in patients with advanced clinical stages (III or IVa) compared to those with early stages (I or II). It was hypothesized that GNA14 may function as an oncogene, with low expression promoting the development and progression of NPC. Strategies to restore GNA14 expression in NPC cells may have the potential to inhibit tumor growth and metastasis. This restoration can be achieved through gene therapy techniques, such as viral or nanoparticle-mediated delivery of GNA14, with the aim of re-establishing its expression within tumor tissues. Furthermore, we focused on DEGs in patients with high and low GNA14 expression. The analysis of GO and KEGG indicates that these DEGs are primarily enriched in pathways associated with immune response and cell migration (30, 31). In addition, GSEA suggested that compared to patients with high GNA14 expression, pathways such as cell division, DNA, and chromosome replication were up-regulated in patients with low GNA14 expression, while pathways such as immune cell activity and adaptive immune response were down-regulated (32). Research has demonstrated that the extent of immune infiltration is linked to prognosis, with greater levels of immune infiltration generally indicating a more favorable prognosis (33). Our study discovered that NPC patients with low GNA14 expression had a significant decrease in the proportion of most immune cells, such as B cells, CD4+, and CD8+ T cells, and had lower stromal scores and immunity scores, indicating these patients had higher tumor purity and lower levels of immune infiltration (22). It is anticipated that the restoration of GNA14 expression or activity will enhance the immune infiltration of tumors and augment the capacity of the immune system to combat tumor growth. The combination of GNA14-targeted therapy with immune checkpoint inhibitors (e.g., PD-1/PD-L1) may result in a synergistic effect and an improvement in therapeutic efficacy. In addition, we found that patients with low GNA14 expression were less sensitive to chemotherapeutic agents such as 5-fluorouracil and gemcitabine, which are commonly used chemotherapeutic agents in nasopharyngeal carcinoma, suggesting that these patients may have poorer response to chemotherapy.

Numerous studies have demonstrated a close association between EBV infection and the development of NPC. EBV DNA can be used as a biomarker for early diagnosis and prognostic prediction of NPC (34, 35). In our study, multivariate Cox regression analysis showed that pre-treatment EBV DNA was an independent risk factor for PFS and DMFS in patients, which is consistent with the findings of Tang et al. (36). In addition to EBV DNA, many biomarkers have been reported to be associated with the prognosis of NPC, such as EBV serum antibodies, miR-BART2-5p, serum LDH, and C-reactive protein (CRP) (37–40). These factors have limitations in clinical application due to their susceptibility to patient conditions and the complexity of the inspection techniques. We found that GNA14 expression and N stage were equally independent risk factors for DMFS. We have developed a nomogram based on GNA14 expression that can more easily help clinicians evaluate patients with a high risk of distant metastasis. For instance, a more intense regimen can be used in individuals with low GNA14 expression.

Our study had some limitations. First, this study was a single-center study, and all patients were enrolled in a high-prevalence area, thus not able to generalize to all NPC patients. It is recommended that future studies integrate data from different sources and a broader patient population for multicenter validation. Second, due to practical constraints, we were unable to collect data from a larger number of participants. A small sample size may reduce the statistical power to detect differences. However, if the results demonstrated statistical significance under these circumstances, it emphasizes the robustness of the findings. Third, immune cell infiltration and drug sensitivity analysis with GNA14 expression has not been fully validated in cellular or other experimental studies. Future studies are needed to elucidate the precise role of GNA14 in nasopharyngeal carcinogenesis, invasion, and metastasis, along with its impact on immune cell infiltration and drug sensitivity, through experiments like *in vitro* cellular assays and animal models.

## Conclusion

In conclusion, we found that GNA14 expression was down-regulated in NPC tissues, and its low expression may be closely associated with advanced tumor stage and distant metastasis. GNA14 expression combined with pre-treatment EBV DNA load and N stage shows potential for predicting NPC patients with a high risk of distant metastasis.

## Data Availability

The original contributions presented in the study are included in the article/**Supplementary Material**. Further inquiries can be directed to the corresponding authors.

## Supplementary material

Supplementary Table 1 The characteristics of the GEO datasets and the tumor types in the TCGA database.

Supplementary Table 2 The results of differential expression analysis in the training set.

Supplementary Table 3 Results of RF algorithm, LASSO regression algorithm, and SVM-RFE algorithm.

Supplementary Table 4 The DEGs between high and low GNA14 expression groups in GSE102349.

Supplementary Table 5 List of immune checkpoint genes highly correlated with GNA14.
